# Intra-Procedural Real-Time Predictors of Failure in Patients with Roux-en-Y Gastric Bypass Undergoing Double-Balloon Assisted ERCP: Is There an Optimal Time to Cross-Over to EUS-Directed Transgastric ERCP? A Prospective Single-Center Study

**DOI:** 10.3390/jcm15020765

**Published:** 2026-01-17

**Authors:** Kambiz Kadkhodayan, Azhar Hussain, Saurabh Chandan, Shayan Irani, Almujarkesh Mohamad Khaled, Abdullah Abbasi, Mustafa Arain, Natalie Cosgrove, Maham Hayat, Deepanshu Jain, Sagar Pathak, Dennis Yang, Zubair Khan, Armando Rosales, Hasan K. Muhammad

**Affiliations:** 1 AdventHealth, Orlando, FL 32803, USA; kamkad@gmail.com (K.K.); azharhussain0139@gmail.com (A.H.); mohamadkhaled.almujarkesh.md@adventhealth.com (A.M.K.); mustafa.arain.md@adventhealth.com (M.A.); natalie.cosgrove.md@adventhealth.com (N.C.); maham.hayat.md@adventhealth.com (M.H.); deepanshu.jain.md@adventhealth.com (D.J.); dennis.yang.md@adventhealth.com (D.Y.); zubairkhan254@gmail.com (Z.K.); armando.rosales.md@adventhealth.com (A.R.); muhammad.hasan.md@adventhealth.com (H.K.M.); 2 Houston Methodist West Hospital, 18300 Katy Fwy Suite 425, Houston, TX 77094, USA; 3 Virginia Mason Medical Center, Seattle, WA 98101, USA; shayan.irani@virginiamason.org; 4 Department Gastroenterology, University of Iowa, Iowa City, IA 52242, USA; abdullahabbasi19@gmail.com; 5 Department Gastroenterology, Loma Linda University, Loma Linda, CA 92354, USA; sagar.j.pathak@gmail.com

**Keywords:** EDGE, double-balloon enteroscopy, endoscopic retrograde cholangiopancreatography

## Abstract

**Background:** Endoscopic retrograde cholangiopancreatography (ERCP) in patients with Roux-en-Y gastric bypass (RYGB) remains technically challenging. Device-assisted ERCP (DAE-ERCP) is widely used for uncomplicated pancreaticobiliary disease but is associated with prolonged procedure times and high failure rates. Endoscopic ultrasound-directed transgastric ERCP (EDGE) offers high technical success but introduces additional cost and the risk of a persistent fistula. We aimed to prospectively identify intra-procedural predictors of DAE-ERCP failure and define an actionable, real-time threshold for early cross-over to EDGE. **Methods:** We prospectively evaluated consecutive RYGB patients undergoing DAE-ERCP at a tertiary referral center. Patients with established pre-procedural features associated with complex or low-yield DAE-ERCP were triaged directly to EDGE and excluded. Intra-procedural variables were recorded in real time. Univariate and multivariable logistic regression identified predictors of DAE-ERCP failure. Received operating characteristic (ROC) analysis determined optimal cutoffs for cross-over. **Results:** A total of 94 patients with RYGB underwent ERCP. Amongst these, 42 patients (11 males, 31 females) were included in the analysis and underwent DAE-ERCP with a success rate of 73.8% (n = 31). Significant risk factors of DAE-ERCP failure included excessive resistance to scope advancement (*p* < 0.0001), failure to reach the ampulla (*p* < 0.0001), patient position (*p* = 0.009), BMI (*p* = 0.004), and time to reach the jejuno-jejunal (J-J) anastomosis (*p* < 0.0001). Additionally, time needed to reach the J-J anastomosis of ≥23 min [OR 1.360 (95% CI: 1.079–1.713), *p* = 0.009], excess resistance to scope advancement [OR 2.223 (95% CI: 2.001–4.167)], and failure to reach the ampulla [OR 9.929 (95% CI: 2.004–4.033)] were statistically significant predictors of DAE-ERCP failure. When ≥2 predictors of BA-ERCP failure were present, the likelihood of DAE-ERCP failure was 2.370 with 95.50% sensitivity and 90% specificity with AUC= 0.929 (*p* = 0.0001). **Conclusions:** DAE-ERCP remains an effective first-line strategy in appropriately selecting RYGB patients without pre-procedural high-risk features. Real-time intra-procedural predictors can reliably identify impending failure. A structured algorithm incorporating both pre-procedural triage and intra-procedural checkpoints supports timely transition to EDGE, optimizing efficiency, safety, and resource utilization.

## 1. Background

The global prevalence of obesity has increased steadily over the past decade, with projections suggesting that nearly half of adults in the United States will meet criteria for obesity by the year 2030 [1]. Bariatric surgery remains one of the most effective interventions for sustained weight loss and improvement in metabolic comorbidities, and Roux-en-Y bypass (RYGB) continues to be amongst the most widely performed weight loss operations [2]. With an increase in the number of post-RYGB patients, gastroenterologists are increasingly confronted with pancreaticobiliary disease in the setting of surgically altered anatomy.

Gallstone disease and choledocholithiasis are substantially more prevalent in patients with obesity, and the rapid weight loss that follows bariatric surgery further increases the risk of biliary stone formation [3,4]. As a result, there is a growing number of RYGB patients that require ERCP for indications ranging from uncomplicated biliary stones to more complex conditions like biliary strictures and biliopancreatic malignancy. ERCP in this population is fundamentally constrained by exclusion of the native stomach and duodenum, elongation of the alimentary and biliopancreatic limbs, and sharp angulations at the jejuno-jejunal (JJ) anastomosis [5,6,7].

Historically, these anatomic challenges have required surgical or percutaneous approaches to biliary drainage, both of which are associated with increased morbidity, longer hospital stays, and higher healthcare costs [8]. Several alternative strategies have been developed to address this challenge, including laparoscopic-assisted ERCP (LA-ERCP), device-assisted ERCP (DAE-ERCP), and endoscopic ultrasound-directed transgastric ERCP (EDGE) [9,10,11,12]. Each approach offers distinct advantages and limitations, and an optimal strategy remains the matter of ongoing debate. DAE-ERCP preserves post-surgical anatomy and avoids surgical or transmural access, but is technically demanding, time-intensive, and is associated with lower overall success rates when compared to EDGE [13].

The development of device-assisted enterostomy, particularly double-balloon enteroscopy, represented a major advancement, by enabling deep intubation of the small bowel and retrograde access to the ampulla in patients with altered anatomy [14,15]. Subsequent milestones including the development of a short-type enteroscope with a larger working channel, have improved accessory compatibility and expanded the therapeutic capabilities of DAE-ERCP [16,17]. Despite these advances, DAE-ERCP remains technically demanding and is highly operator dependent. Reported technical success rates vary widely, and failure is not uncommon, even in experienced hands. Prolonged procedure times, excessive looping, bowel adhesions, and unfavorable papillary orientation, frequently limit successful deep cannulation of the bile duct and therapy. Importantly, DAE-ERCP failure frequently results in an aborted procedure, repeat anesthesia exposure, and delayed definitive management [18,19,20,21].

In recent years, EDGE has emerged as a viable endoscopic alternative for RYGB patients. By creating a temporary gastro-gastric (GG) or jejuno-gastric (JG) fistula using a lumen-apposing metal stent, EDGE restores native access to the ampulla, allowing the use of a standard duodenoscope and a broader range of ERCP accessories [22]. Multiple comparative studies and meta-analyses have demonstrated higher technical and clinical success rates with EDGE when compared to DAE-ERCP, particularly for complex biliary disease [23,24]. Despite its many advantages, EDGE introduces additional procedural steps, requires transmural access, is associated with stent-related adverse events such as migration and persistent fistula formation and frequently mandates a staged approach, involving more than one procedure [13,22,25].

In contemporary Western practice, based on available expertise, many centers continue to attempt DAE-ERCP as a first-line approach for patients with uncomplicated biliary disease, reserving a separate-session EDGE procedure or LA-ERCP as a salvage strategy after DAE-ERCP failure. This approach, while intuitive, will often result in prolonged procedures and inefficient care, as the decision to abandon DAE-ERCP is frequently made later and subjectively. There is a paucity of prospective data defining real-time intra-procedural thresholds beyond which continued attempts at DAE-ERCP are un-likely to succeed [25].

There are several studies that have identified pre-procedural factors that are associated with lower success rates for DAE-ERCP. These include malignant biliary obstruction, need for advanced pancreatic or biliary intraductal therapy and large or complex bile duct stones [26,27,28,29]. Despite this, many centers continue to attempt delayed DAE-ERCP broadly, often resulting in prolonged procedures, aborted cases, repeat anesthesia exposure, increased healthcare costs, and delays in definitive therapy.

Even amongst carefully selected patients, there remains a critical unmet need for objective intra-procedural criteria to guide timely abandonment of DAE-ERCP and transition to alternative modalities such as EDGE. Prolonged attempts in the face of diminishing returns increase the risk of adverse events without improving the likelihood of success.

We therefore adopted a selective, two-stage algorithmic strategy in which DAE-ERCP was reserved exclusively for patients without established pre-procedural predictors of failure, followed by real-time intra-procedural assessment to guide early cross-over to EDGE when predicted thresholds are met. This prospective study evaluates intra-procedural predictors of DAE-ERCP failure within this framework and promotes a unified decision-making algorithm for ERCP in patients with RYGB.

## 2. Methods

This prospective, single-center study was conducted at a tertiary referral center specializing in therapeutic endoscopy. Consecutive adult patients with prior RYGB referred for endoscopic management of pancreaticobiliary disease were screened for inclusion between 2022 and 2024. The study protocol was approved by our institutional review board, and the requirement for individual informed consent was waived due to the observational nature of the study.

At our institution, patients with RYGB anatomy and pancreaticobiliary disease are preferentially referred for endoscopic evaluation when endoscopic therapy is considered feasible based on clinical stability, anatomy, and anticipated therapeutic requirements. Percutaneous transhepatic cholangiography is typically reserved for patients with failed endoscopic access, hemodynamic instability, or in situations where endoscopic intervention is not feasible. This referral pattern reflects our institutional practice and was not altered for purposes of this study.

All referred patients underwent standardized pre-procedural evaluation including a detailed clinical history, review of operative reports when available, cross-sectional imaging, and laboratory assessment. Imaging was used to confirm the indication for intervention, access stone burden and bile duct anatomy, and exclude features that suggest complex disease that may require advanced intraductal therapy. Patients with pre-procedural features associated with low procedural yield for DAE-ERCP were triaged to alternative approaches and excluded from the analysis. Based on existing literature and institutional experience, DAE-ERCP was attempted in patients without complex malignant biliary obstruction, primary pancreatic ductal indications, large or complex bile duct stones (defined as stones ≥ 15 mm, impacted stones, intrahepatic stones, or stones associated with distal strictures), anticipated need for advanced accessories such as cholangioscopy or large-caliber metal stents, and clinical features suggestive of severe adhesive disease or complex open abdominal surgery.

**Figure 1:**
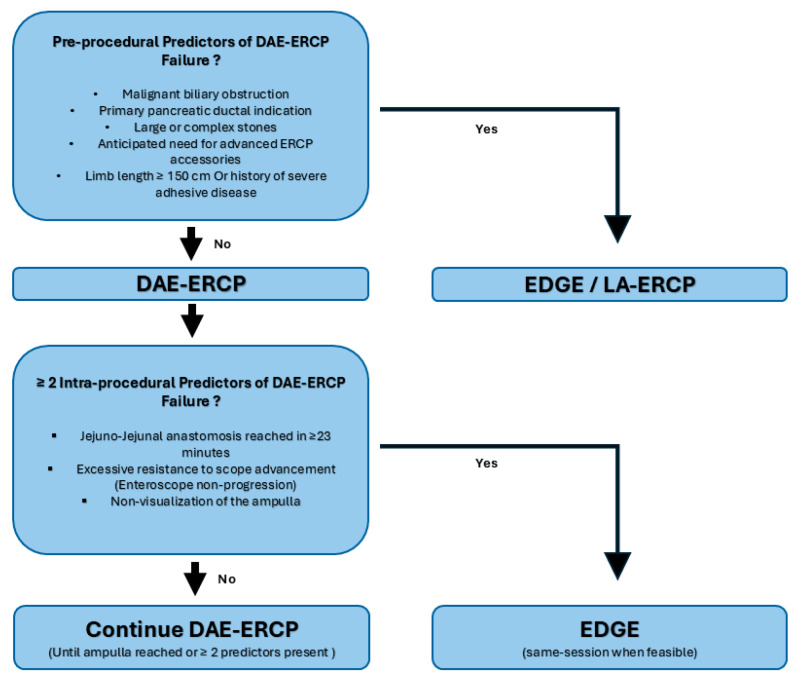
Algorithm used for ERCP in patients with Roux-en-Y gastric bypass.

Patients meeting any of these criteria were triaged directly to EDGE or alternative approaches, including LA-ERCP or EUS-guided biliary drainage, depending on clinical urgency and institutional resources. Only patients deemed appropriate for DAE-ERCP based on this selective strategy, including patients with simple distal biliary strictures in the setting of previously diagnosed malignancy, were included in the present analysis (Figure 1).

DAE-ERCP was performed using a double-balloon enteroscopy system, preferentially employing a short-type enteroscope to maximize accessory compatibility. All procedures were performed by six experienced therapeutic endoscopists (KK, DY, MA, MH, DJ, NC) with extensive expertise in altered anatomy ERCP. All procedures were performed under general anesthesia. Patients were positioned in the left lateral decubitus position or the prone position, based on operator preference. When performing DAE, enteroscope advancement was performed using standard push-and-pull techniques with attention to loop reduction and avoidance of excessive force. The selective application of gentle abdominal pressure was used if and when needed. All procedures were performed by attending physicians with extensive experience in ERCP in surgically altered anatomy. While trainees were present in the majority of cases, most procedural steps including enteroscope advancement, cannulation, and therapeutic decision-making, were performed by the attending physician.

Intra-procedural variables were prospectively recorded in real time, including time to reach the JJ anastomosis, subjective assessment of resistance to scope advancement or scope non-progression, ability to reach and visualize the ampulla, and total procedure duration. Time to reach the JJ anastomosis was defined as the elapsed time, in minutes, from scope insertion at the lip-line, to endoscopic visualization of the JJ anastomosis. This time interval was recorded by assisting staff and was intended to reflect the cumulative impact of limb length, angulation, and looping as well as the effect of intra-abdominal adhesions on enteroscope advancement. Excessive resistance to scope advancement was defined as the presence of fixed, non-yielding resistance to enteroscope advancement despite standard loop-reduction maneuvers, balloon manipulation, patient repositioning, and gentle abdominal pressure. Resistance was considered excessive when forward advancement required sustained forward force or resulted in repeated scope recoil, prompting concern for increased risk of traction-related injury or low likelihood of further progression. This variable was assessed by the performing endoscopist and recorded in real time by the support staff. Failure to reach the ampulla was defined as the inability to visualize or access the ampulla despite systematic scope advancement and reasonable procedural effort as determined by the performing endoscopist.

Technical success was defined as deep cannulation of the intended duct with completion of the planned therapeutic intervention during the index procedure. Procedural failure was defined as the inability to achieve these endpoints, including failure to reach the ampulla, inability to cannulate the intended duct or abandonment of the procedure due to unfavorable intra-procedural findings. Clinical success was defined as resolution of the index biliary indication following the index procedure or planned staged therapy, as evidenced by improvement or normalization of cholestatic liver enzymes, resolution of biliary obstruction-related symptoms, and the absence or need for urgent re-intervention during the index hospitalization. In patients undergoing biliary stent placement due to incomplete duct clearance or strictures, clinical success was assessed based on symptomatic and biochemical improvement rather than immediate complete duct clearance. Patients who required cross-over to EDGE for definitive therapy after DAE-ERCP were not considered to have achieved clinical success with DAE-ERCP.

Continuous variables were compared using *t*-tests or Wilcoxon rank sum tests and categorical variables were analyzed using chi-squared or Fisher’s exact tests. Significant univariate predictors were further analyzed using binary logistic regression and ROC curves determining optimal cross-over timing. Given the prospective design and selective inclusion strategy, no formal power analysis was performed. Statistical significance was set at *p* < 0.05.

## 3. Results

A total of 94 patients with RYGB underwent ERCP during the study period. Amongst these, 42 patients (mean age 58.5 ± 12.9 years; 26.2% male) underwent DAE-ERCP. Success was achieved in 31 patients (73.8%), while 11 (26.2%) experienced failure. The ampulla was not reached in 26.1% (n = 11) of cases. On univariate analysis, successful DAE-ERCP was significantly associated with lower BMI (29.6 ± 7.4 vs. 39.4 ± 12.3, *p* = 0.004) and a shorter time to reach the J-J anastomosis (5.3 ± 2.8 vs. 28.5 ± 12.3 min, *p* < 0.0001). Excessive resistance to scope advancement (*p* < 0.0001), failure to reach the ampulla (*p* < 0.0001), and patient position (*p* = 0.009) were also found to be statistically significantly different (Table 1).

Binary logistic regression analysis identified the time to reach the J-J anastomosis ≥ 23 min [OR 1.360 (95% CI: 1.079–1.713), *p* = 0.009], excessive resistance to scope advancement [OR 2.223 (95% CI: 2.001–4.167)], and failure to reach ampulla [OR 9.929 (95% CI: 2.004–10.033)] as the statistically significant predictors of DAE-ERCP failure (Table 2).

**Figure 2:**
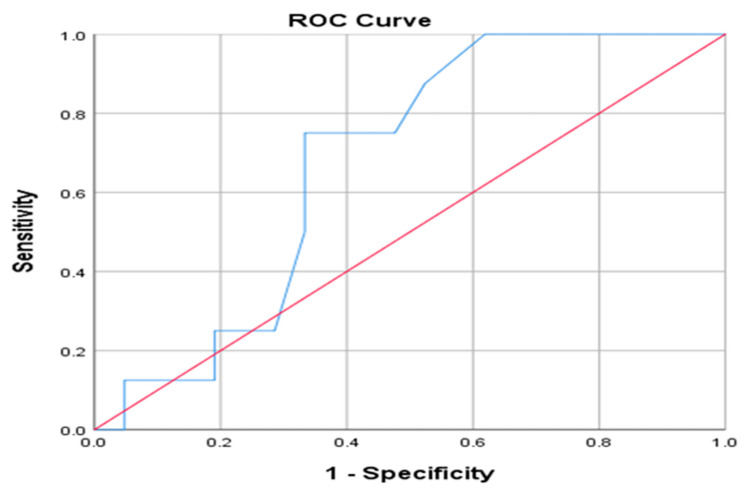
ROC curve for predicting failure based on time to J-J anastomosis. Red: Diagonal line for random classifier. Blue: Area Under the Curve (AUC).

On ROC curve analysis, a threshold of ≥23 min to reach the J-J anastomosis predicted procedure failure with 90% sensitivity and 79% specificity (AUC = 0.723, *p* < 0.0001) (Figure 2).

**Figure 3:**
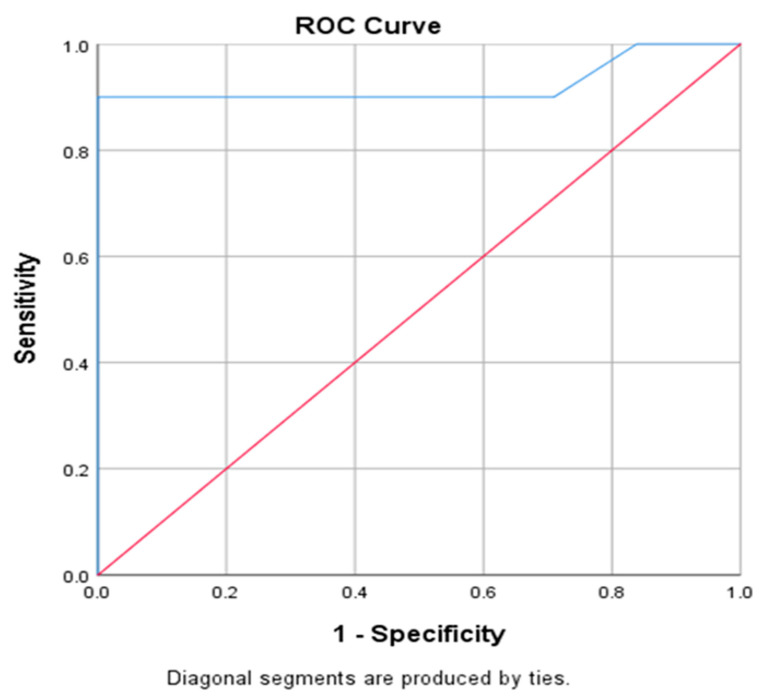
ROC curve for predicting failure based on ≥2 predictors of DAE-ERCP failure. Red: Diagonal line for random classifier. Blue: Area Under the Curve (AUC).

When there were ≥2 predictors of DAE-ERCP failure, the odds of having failed DAE-ERCP were 9.6 with 95.50% sensitivity and 90% specificity with AUC= 0.929 (*p* = 0.0001) (Figure 3).

## 4. Discussion

This prospective study provides evidence to support a structured, algorithmic approach to ERCP in patients with Roux-en-Y gastric bypass anatomy. By integrating deliberate pre-procedural patient triage with real-time intra-procedural predictors of failure, we demonstrate that DAE-ERCP failure can be anticipated during the procedure with high accuracy. The principal finding of this study is that prolonged time to reach the JJ anastomosis, excessive resistance to scope advancement, and failure to reach or visualize the ampulla are strong complementary predictors of failure, and that the presence of two or more of these features defines an actionable point to abandon DAE-ERCP early and transition to EDGE in the same session.

An important aspect of this study was the deliberate restriction of DAE-ERCP to patients without established pre-procedural predictors of failure. There are ample examples in the literature that have consistently demonstrated that DAE-ERCP performs best in patients with uncomplicated biliary disease requiring limited intervention, while outcomes are inferior in patients requiring advanced therapy or repeated access. In contrast, EDGE offers consistently high technical and clinical success across a broad range of indications, particularly in patients with complex disease, though at the cost of higher resource utilization and stent-related risks. By triaging patients with suspected malignancy, large or impacted stones, anticipated need for cholangioscopy or lithotripsy, or expected need for repeated ERCP directly to EDGE, we aimed to avoid predictable low-yield DAE-ERCP attempts. This selection strategy reflects a shift away from a “one size fits all” approach to a more selective choice based on disease complexity and anticipated therapeutic needs.

Amongst the intra-procedural predictors identified, time to reach the jejuno-jejunal anastomosis emerges as a particularly valuable early marker. This variable reflects the cumulative effect of limb length, angulation, presence of adhesions, and looping of the scope, all of which directly impact the likelihood of reaching the biliopancreatic limb and ampulla. Prior studies have reported prolonged procedure times and lower success rates in similar settings, but none have proposed a concrete, time-based threshold for decision-making. A threshold of 23 min in our cohort provided high sensitivity for predicting failure and allowed the identification of difficult cases before excessive procedure time or operator fatigue set in. It is important to emphasize that this threshold is not intended to be rigid, but rather to serve as an objective prompt for procedure feasibility assessment. In practice, early recognition of inadequate progression allows the endoscopists to reassess and possibly shift strategy before committing to prolonged, low-yield attempts.

Excessive resistance to scope advancement was observed in a high fraction of failed cases. Fixed resistance often reflects intra-abdominal adhesions, internal hernias, or sharp angulation and may predispose to traction associated perforation if scope advancement is forced. Recognition of this feature requires user experience and clinical judgment, but its presence should prompt caution and reconsideration of continued enteroscope advancement. While the importance of avoiding excessive fixed resistance is well established in enteroscopy literature, it has not been well studied in the context of DAE-ERCP, where avoiding excessive forward pressure on the enteroscope may be of particular importance given the high likelihood of having post-operative adhesions and a higher risk of traction-related perforations. Our data suggests that fixed resistance to progression of the enteroscope should not be viewed as a technical limitation, but as an independent predictor of failure.

Failure to reach or visualize the ampulla represents a late but decisive predictor of DAE-ERCP failure. Once systematic limb exploration has been performed and ampullary access remains out of reach, the likelihood of success diminishes substantially. Persisting beyond this point often results in diminishing returns and increased risk without meaningful benefit. While this study focuses on predictors of failure, it does not diminish the importance of technical excellence in DAE-ERCP. Careful limb identification, judicious insufflation, appropriate patient positioning, and fluoroscopic confirmation of enteroscope orientation remain essential components of safe and effective practice. Our findings emphasize that while technical optimization should be completed with pre-defined thresholds for procedure abandonment. Technical mastery improves overall success but cannot overcome fundamentally unfavorable anatomical factors.

Comparative studies and meta-analyses consistently demonstrate higher success rates for EDGE when compared to DAE-ERCP, particularly in patients with complex disease. Our findings do not contradict this evidence but rather refine its application. We propose a hybrid strategy that leverages the strengths of both modalities by reserving EDGE for patients with pre-procedural high-risk features and for those who meet intra-procedural failure criteria during DAE-ERCP.

Our proposed algorithm begins with pre-procedural assessment to determine candidacy for DAE-ERCP. Patients without predictors of failure undergo DAE-ERCP with prospective monitoring of intra-procedural variables. Time to jejuno-jejunal anastomosis, resistance to enteroscope advancement, and ampullary visualization are accessed continuously. When two or more predictors are present, the procedure is aborted and EDGE is performed during the same session when feasible (Figure 1). This proposed structured approach reduces variability, supports shared decision-making, and provides a defensible framework for same-session procedural transition from DAE-ERCP to EDGE.

Our study has several limitations that merit consideration. First, this was a prospective, single-center observational study, conducted at a large tertiary referral center. This may limit generalizability to centers with different referral patterns and limited technical expertise. Second, our sample size was modest due to the relatively low proportion of RYGB patients that meet our institution’s selection criteria for DAE-ERCP. Third, all procedures were performed by highly experienced therapeutic endoscopists with substantial expertise in both DAE-ERCP and EDGE. This limits extrapolation to centers with lower procedural volumes. Finally, although the intra-procedural variables were prospectively defined and recorded in real-time, certain elements such as excessive resistance to scope advancement remain partially subjective and operator dependent. The intra-procedural predictors identified in this study should therefore be viewed as guidance points, rather than ridged rules, particularly in settings where operator expertise differs.

## 5. Conclusions

In patients with Roux-en-Y gastric bypass anatomy, DAE-ERCP remains an effective first-line approach when pre-procedural predictors of failure are absent. Real-time intra-procedural predictors, particularly a prolonged time to jejunal-jejunal anastomosis of ≥23 min, excessive resistance to scope advancement, and failure to reach the ampulla, reliably identify impending failure. The presence of two or more of these predictors represents an optimal threshold for early cross-over to EDGE. Adoption of a structured, algorithmic approach that integrates pre-procedural selection and intra-procedural decision-making, has the potential to improve efficiency, safety, reduce healthcare utilization and potentially improve patient outcomes.

## Figures and Tables

**Table 1:** Clinical and intra-procedural predictors of DAE-ERCP outcomes.

Variable	Successful DAE-ERCP (n = 31)	Failed DAE-ERCP (n = 11)	*p*-Value
**Gender, %** Female Male	23 (74.20%) 8 (25.80%)	8 (72.70%) 3 (27.30%)	0.924
**Age (years), mean ± SD**	58.94 ± 13.67	57.36 ± 11.18	0.734
**Body mass index (BMI), kg/m^2^**	29.60 ± 7.37	39.36 ± 12.33	**0.004**
**Indication for procedure, %** PD leak Malignancy Other Choledocholithiasis Acute cholangitis Bile leak	1 (3.20%) 2 (6.50%) 7 (22.60%) 15 (48.40%) 5 (16.10%) 1 (3.20%)	0 3 (27.30%) 3 (27.40%) 4 (36.60%) 0 1 (09.10%)	0.306
**Excess resistance to scope advancement, %** Yes No	0 21 (100.0%)	6 (54.50%) 5 (45.50%)	**<0.0001**
**Patient position during procedure, %** Left lateral Prone	28 (90.30%) 3 (9.70%)	6 (54.50%) 5 (45.50%)	**0.009**
**Time since RYGB surgery (years), mean ± SD**	12.88 ± 8.71	16.60 ± 15.38	0.629
**Time to reach JJ anastomosis (mins), mean ± SD**	5.29 ± 2.79	28.50 ± 12.29	**<0.0001**
**Time from JJ anastomosis to ampulla (mins), mean ± SD**	11.97 ± 8.31	17.00 ± 13.42	0.556
**Ampulla reached** Yes No	31 (100.0%) 0	1 (9.10%) 10 (90.90%)	**<0.0001**
**Total time for scope to reach papilla (mins), mean ± SD**	18.00 ± 9.66	20.00 ± 8.76	0.839
**Total procedure time (mins), mean ± SD**	59.68 ± 28.02	77.50 ± 22.50	0.069

**Table 2:** Logistic regression analysis of intra-procedural predictors of DAE-ERCP failure.

Predictor	Odds Ratio (95% CI)	*p*-Value
Time to reach J-J anastomosis ≥23 min	1.360 (1.079–1.713)	**0.009**
Excess resistance to scope advancement	2.223 (2.001–4.167)	**0.021**
Patient position during procedure	0.218 (0.001–8.887)	0.594
Failure to reach ampulla	9.929 (2.004–4.033)	**0.005**

## Data Availability

Data is available at reasonable request after approval from AdventHealth Orlando hospital.
